# Education Research: The Current Landscape of Clinician Educator Tracks in Adult Neurology Residency Programs: A National Survey of Program Directors

**DOI:** 10.1212/NE9.0000000000200142

**Published:** 2024-08-06

**Authors:** Nuri Jacoby, K.H. Vincent Lau, Maureen I. Ekwebelem, Jeremy J. Moeller, Daniel Shalev

**Affiliations:** From the Department of Neurology (N.J.), SUNY Downstate Health Sciences University; Department of Neurology (N.J.), Maimonides Medical Center, Brooklyn, NY; Department of Neurology (K.H.V.L.), Boston University School of Medicine, MA; Division of Geriatrics and Palliative Medicine (M.I.E., D.S.), Weill Cornell Medicine, New York, NY; and Department of Neurology (J.J.M.), Yale School of Medicine, New Haven, CT.

## Abstract

**Background and Objectives:**

As the concept of a clinician-educator (CE) evolves and the multiple competencies of the role become better defined, there seems to be a growing need for targeted training for clinicians pursuing a career in medical education. This study aims to describe the current state of CE tracks in adult neurology residency programs and to identify the barriers to implementation, potential solutions, and program goals and outcomes.

**Methods:**

We characterized CE tracks using 2 methods. First, we reviewed the websites of all US adult neurology residency programs to determine the availability of a CE track and its characteristics. Second, we administered a 20-item survey to program directors (PDs) of all US neurology residency programs, with questions focused on track availability, characteristics, perceived benefits of CE tracks on resident career development, barriers to implementation, and ideas for national initiatives that may facilitate track development or improvement.

**Results:**

Fifty-eight of 177 (33%) PDs responded to the survey. Combining the results of the website reviews and surveys, we found that 34 of 179 (19%) programs have CE tracks. Seventy percent of PDs felt that CE tracks are very impactful or impactful for participating residents' careers, a perception more common among PDs of programs with tracks. The greatest perceived benefit was in preparing residents for educational leadership roles. The greatest barriers to implementation were a lack of teaching faculty, a lack of resources, and limited resident time. The highest ranked idea for a national initiative that can facilitate track development was live and recorded lectures on medical education topics.

**Discussion:**

Although most PDs surveyed agreed that CE tracks are impactful for preparing residents as teachers and education leaders, such tracks are available in only 19% of adult neurology residency programs. PDs report that the benefits of CE tracks extend beyond the participants, with implications for the health of the residency program and the neurology department. While some programs have significant barriers to implementation, national initiatives may help reduce the resource burden on individual programs. Future areas of study include assessing the development and outcomes of national initiatives and analyzing the outcomes associated with CE tracks.

The clinician-educator (CE) concept has significantly evolved since its initial introduction, likely in the 1980s.^1^ One of its earliest definitions was a superior clinician who is also a dedicated teacher.^2^ The role has grown in scope since; teaching skills are only one of multiple competencies comprising a CE's professional identity. The various dimensions of the CE role are highlighted by the recently published CE milestones,^3^ co-developed by the Accreditation Council for Graduate Medical Education (ACGME), Association of Medical Colleges, and American Association of Colleges of Osteopathic Medicine. The CE milestones are categorized into 5 competencies: universal pillars; administration; diversity, equity, and inclusion; well-being; and educational theory and practice. Unlike the specialty-specific milestones used to evaluate trainees, these milestones are meant to be used as a self-assessment tool for faculty educators and educational scholars; they have no role in program ACGME accreditation.^4^ While the CE milestones help operationalize a CE's roles, these roles continue to evolve. Most recently, 3 distinct subcategories of CEs were described: core teaching faculty who primarily provide clinical care, educators with leadership roles, and educational scholars.^5^

The emergence of the CE as a distinct career track has led to the development of CE tracks for residents interested in a CE career. Residency tracks, whether they are CE, research, or global health tracks, provide residents who have identified specific goals early in their training with tailored longitudinal educational experiences.^6^ CE tracks were first described in 3 psychiatry programs in 2010, followed by an internal medicine program in 2014.^7,8^ Most CE tracks include a combination of didactic and experiential sessions and a scholarly project. Teaching and scholarship skills are the most frequently addressed topics.^9,10^ Although studies assessing outcome measures of tracks have been limited, residents generally report benefits that include the development of feedback and teaching skills, a clearer understanding of the breadth of CE careers, and networking opportunities within the medical education community.^11^ Although CE tracks have multiple benefits, implementation barriers include a lack of faculty time and expertise, limited resources, and absence of financial support.

Despite the importance of defining the CE role and characterizing CE tracks among all medical specialties, published research has focused on internal medicine,^7,11^ family medicine,^12^ and psychiatry residency programs.^8,13^ This study aims to characterize the current landscape of CE tracks in adult neurology residency programs, understand residency program director (PD) perspectives on and experiences with CE tracks, and explore the perceived benefit of national initiatives to support local CE tracks.

## Methods

### Study Design

This was a 2-part study. The first part was a review of the websites of all US adult neurology residency programs to collect information about CE tracks. The second part was a survey of PDs in which website data were confirmed and PD perspectives on CE tracks were explored in detail.

### Website Reviews

Two authors reviewed all program websites to ensure accuracy. Author ME conducted the first review of the programs, and authors N.J. and K.H.V.L. conducted the second review. Any discrepancies were discussed and clarified by the 2 website reviewers. For all websites, we recorded the overall number of residents per year and whether a CE track or medical education elective was available. We also recorded whether a graduate medical education (GME)-wide CE track not specific to neurology residents was available. If a CE track was present, we then reviewed the website for 7 characteristics: (1) whether residents have to apply to the track; (2) if there is a limit to the number of enrolled residents in each class, and (3) if so, the limit; (4) resources available to residents on the track; (5) track requirements; (6) whether participants received a certificate or another form of recognition upon completion; and (7) who the track director is. Items were only marked as present if the information was found on the website; otherwise, it was marked as “unknown.”

### Survey Design

Given the lack of a validated survey appropriate to our study topic, we designed a 20-question survey tool (see eTable 1) using the 7-step survey design recommended by AMEE guide number 87.^14^ (1) The survey items were informed by a literature review, which was also conducted to ensure that no existing tool could be adapted. (2) The lead author conducted interviews with 2 CE track directors. (3 and 4) This information was synthesized to develop items. (5) Items were validated by the 2 experts in step 2. (5 and 6) Items were then iteratively pilot-tested on 2 associate PDs to ensure that items were understood as intended. Associate PDs were chosen to pilot the survey because they would share a frame of reference with intended participants but not be potential study participants. The survey was administered using RedCap, a secure, web-based software platform.^15^

Survey items included PD demographics (program, name, email), size of the program, availability of a CE track, availability of a medical education elective for residents, and availability of a GME-wide (not program-specific) medical education track or course, perceived barriers to implementing a track, and perceived impact of CE tracks on resident career development. We also asked survey respondents to rank 8 potential career benefits to residents participating in CE tracks and 8 desired national resources to help programs start or maintain a successful CE track. PDs who answered that they had a CE track were asked about the characteristics of the track, mirroring the checklist used in the website review. PDs who responded that they did not have a track were asked how strongly they would consider creating a CE track in the next 5 years.

### Survey Sample and Data Collection

A list of adult neurology residency programs and PDs was obtained from the FREIDA American Medical Association database^16^ and cross-referenced with the ACGME neurology program database.^17^ During the review of program websites, all discrepancies between the 2 databases were clarified.

### PD Survey Distribution

We used RedCap's automated distribution system to send the survey to PDs on September 27, 2023. Two weeks later, on October 11, 2023, we sent an automated reminder to nonresponders. The first author then sent a second reminder email to nonresponders during the week of October 23, 2023.

### Data Analysis

Descriptive statistics were used to analyze survey results, which are presented as frequencies, percentages, and medians. Logistic regression was used to explore the association between the presence of a CE track and the availability of a medical education elective and a non-neurology–specific, GME-wide CE track or course. This analysis method was also applied to examine the relationship between the existence of a CE track and PDs' evaluations of the impact of CE tracks on career development.

Spearman correlation coefficient was calculated to investigate any potential correlation between the size of the residency class, the presence of a CE track, and the likelihood that a PD intends to develop a CE track within the next 5 years.

Finally, ordinal logistic regression was used to assess the association between the size of residency programs with a CE track and the range of resources (from 0 to 5) available to participants in those tracks.

### Standard Protocol Approvals, Registrations, and Consents

The institutional review board of Weill Cornell reviewed the study and deemed it not to qualify as human subjects research.

### Data Availability

Anonymized data not published within this article will be made available by request.

## Results

The survey was emailed to 179 PDs. We received delivery error messages to 2 programs for which we could not find alternative contact information. Of the 177 programs contacted, 58 responded (33% response rate). Four (7%) respondents had 1–3 residents per year, 20 (34%) had 4–6 per year, 8 (14%) had 7–8 per year, 11 (19%) had 9–10 per year, and 16 (27%) had 11 or more/year. The geographic distribution of responses was New England (7.0%), Middle Atlantic (30.0%), South Atlantic (17.5%), East North Central (17.5%), East South Central (5.3%), West North Central (5.3%), West South Central (7.0%), Mountain West (1.8%), Pacific West (8.8%), and US Territory (1.7).

### Number and Characteristics of CE Tracks

We calculated the number of programs with a CE track by using the PD survey answers for responding programs and program website review for nonresponding programs. Combining the website review and PD survey results, we found that 34 of 179 (19%) programs have a CE track. Twenty-one of 58 (36%) survey respondents indicated having a CE track, while website review identified 27 of 179 (15%). Of 27 programs with websites identifying CE tracks, 15 had PDs who responded to the survey. Five survey respondents indicated the availability of a CE track not found on their program's website. No programs with information about a track on the website answered the survey saying they did not have a CE track. Geographically, there were 6 tracks in New England, 6 in the Middle Atlantic, 5 in the South Atlantic, 4 in East North Central, 2 in East South Central, 5 in West North Central, 2 in West South Central, 0 in Mountain West, and 4 in the Pacific West.

Table 1 presents the characteristics of the 21 identified CE tracks. Fourteen (67%) require applicants to apply to join, 6 (29%) limit the number of participants in each class to 1 or 2 per year, and 16 (76%) programs award residents who complete the track a certificate. The PD runs the track in 4 of 21 (19%) programs, an associate PD in 9 of 21 (43%) programs, and another faculty member in 8 of 21 (38%) programs.

**Table 1:** Program Characteristics of Clinician-Educator Tracks Based on Program Director Survey, n (%)

Program size (residents per year)	
#1–3	0 (0)
#4–6	5 (24)
#7–8	4 (19)
#9–10	6 (29)
11+	6 (29)
Apply to join track?	
Yes	14 (67)
No	7 (33)
Resident limit per year?	
Yes	6 (29)
No	15 (71)
Resources available	
Mentorship	21 (100)
Funding for scholarly project	11 (52)
Protected time	8 (38)
Funding for conference	7 (33)
Track requirements	
Scholarly project	18 (86)
Participate in medical education curriculum	18 (86)
Didactic teaching	16 (76)
Teaching rotation	9 (42)
Other^a^	5 (24)
Certificate upon completion	
Yes	16 (76)
No	4 (19)
Other	1 (5)
Director of track	
Program director	4 (19)
Associate program director	9 (42)
Other faculty	8 (38)

a Responses included participation in a neurology education journal club and didactic teaching through the Graduate School of Education and education committee membership.

Among survey respondents with a CE track, 21 (100%) provide mentorship to residents in the track, 11 (52%) provide funding for a scholarly project, 8 (38%) provide protected time beyond standard elective time, and 7 (33%) provide funding for conference attendance. Eighteen programs (86%) require track participants to complete a scholarly project and participate in the department's medical education curriculum, 16 (76%) require participants to provide didactic teaching to medical students and/or residents, and 9 (43%) require participants to complete a medical education elective rotation. In addition, 2 require participation in a neurology education journal club, 1 requires education committee membership, and 1 requires didactic courses through the Graduate School of Education. There was no correlation between residency class size and the availability of a CE track (*r* = 0.22, *p* = 0.09) or the number of resources available to residents (*p* = 0.796).

Among programs that do not have a CE track, 7/37 (19%) were extremely likely to create 1 in the next 5 years, 11/37 (30%) were somewhat likely, 9/37 (24%) were undecided, 8/37 (22%) were somewhat unlikely, and 2/37 (5%) were extremely unlikely. There was no correlation between residency class size and the likelihood of PDs developing a CE track (*p* = 0.3).

### Non-neurology Tracks or Courses and Medical Education Electives

Twenty-three of 58 (40%) programs reported a non-neurology–specific, GME-wide medical education track or course for residents. There was no relationship between the availability of an institutional GME-wide CE track and a neurology residency CE track (OR 1.7; 95% CI 0.56–4.99, *p* = 0.35). Twenty-two of 58 (38%) programs reported having a CE elective. Programs with a CE track had a nonsignificant trend toward higher odds (OR 2.6; 95 CI 0.86–7.88; *p* = 0.089) of offering a medical education elective to residents.

### Impact of CE Tracks

Twenty of 57 (35%) PDs felt that CE tracks are very impactful for the career development of neurology residents interested in medical education, 20 (35%) indicated they are impactful, 12 (21%) somewhat impactful, 4 (7%) minimally impactful, and 1 (2%) not impactful. The availability of a CE track correlated with PDs' rating of CE tracks as impactful or very impactful (OR 4.09; 95% CI 1.02–16.38, *p* = 0.031).

### Barriers to Implementing a CE Track

When asked which barriers PDs have experienced in considering or directing a CE track, 34 of 58 (59%) reported a lack of faculty time, 32 of 58 (55%) a lack of resources, 30 of 58 (52%) a lack of resident time, 13 of 58 (22%) a lack of expert faculty, 12 of 58 (21%) institutional barriers (support or medical education resources), 11 of 58 (19%) a lack of resident interest, and 7 of 58 (12%) a lack of faculty interest. Ten (17%; 6 with a CE track and 4 without) PDs reported no barriers (Figure).

**Figure:**
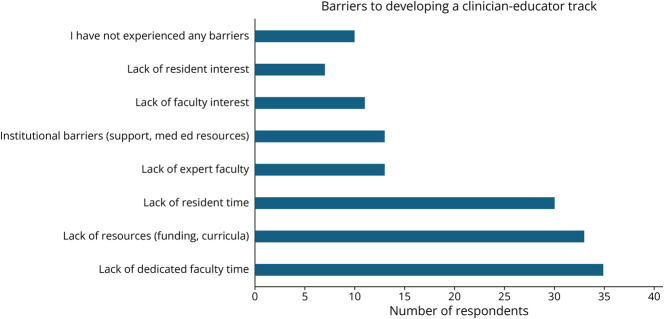
Barriers to Developing a Clinician Educator Track

### Outcomes of CE Tracks

When asked to rank potential outcomes of CE tracks from most to least important, participants responded that many choices were important, with all options chosen as a top 3 rank by at least 12 PDs (Table 2). Preparing residents for administrative medical education leadership roles was the most common top 3 selection, selected by 29 PDs. This was followed by increasing resident satisfaction with residency (top 3 by 25 respondents), promoting resident recruitment (24 respondents), responding to residents' learning goals (22 respondents), and improving the quality of neurologic education in the department (20 respondents). Cultivating national leaders in neurologic education (top 3 by 14 participants), improving the quality of neurologic education nationally (13 participants), and increasing departmental medical education scholarship (12 participants) were considered relatively less important.

**Table 2:** Frequency of Potential Outcomes of Clinician-Educator Tracks Selected as Top 3 Importance, n (%)

Preparing residents for administrative medical education leadership roles	29 (50)
Increasing resident satisfaction with residency experience	25 (43)
Promoting resident recruitment	24 (41)
Responding to resident's learning goals	22 (38)
Improving quality of neurologic education in department	20 (34)
Cultivating national leaders in neurologic education	14 (24)
Improving quality of neurologic education nationally	13 (22)
Increasing departmental medical education scholarly productivity	12 (21)

### Helpfulness of National Initiatives

When asked to rank desired national resources to help PDs' development or maintenance of a CE track, the top 2 options were live and recorded lectures for residents on important topics in medical education (top 3 by 29 respondents) and faculty development seminars that provide guidance and education for implementing a track (23 respondents) (Table 3). The remaining options were ranked relatively similarly, with all chosen as a top 3 rank by at least 17 PDs. These included national guidelines for developing a CE track in neurology (top 3 by 20 respondents), increased grant opportunities in neurology education for faculty (20 respondents), opportunities for residents to become involved in medical education committees or workgroups through national organizations (18 respondents), national CE initiatives that provide mentorship for interested residents (18 respondents) and interested faculty (17 respondents), and increased grant opportunities in education research for trainees (17 respondents).

**Table 3:** Frequency of National Initiatives to Support Local Clinician-Educator Tracks as Top 3 Helpfulness, n (%)

Live and recorded lectures on medical education topics	29 (50)
Faculty development seminars that provide guidance for track implementation	23 (40)
National guidelines for developing a clinician-educator track	20 (34)
Increased grant opportunities in neurology education for faculty	20 (34)
Opportunities for involvement in national committees	18 (31)
Initiative that provides mentorship for interested residents	18 (31)
Initiative that provides mentorship for interested faculty	17 (29)
Increased grant opportunities in neurology education for residents	17 (29)

## Discussion

There is a broad range of availability and specific features of CE tracks in adult neurology residency programs in the United States. Most of the PDs in our study perceive CE tracks as impactful or very impactful, although they are currently available in only 19% of adult neurology residency programs. This discrepancy could be partially explained by the relative novelty of the CE role as a distinct career, such that CE tracks may become increasingly common in the next few years. This is corroborated by our finding that approximately half of the PDs without a CE track plan to implement one in the next 5 years. However, it could also be explained by the identified barriers to implementing a CE track, chief among these the lack of faculty time and institutional support, as reported by half of the survey respondents. PDs may be hesitant to develop a CE track because they do not feel they have the administrative time to direct the track themselves. Our survey found, however, that in 81% of programs, the faculty member running the track was not the PD, which emphasizes that programs, especially in larger departments, can leverage other faculty to take a leadership role running a track.

Many of the benefits of a CE track are intuitive, particularly those related to improved teaching skills among residents and career development opportunities, the latter of which was shown in a prior study of graduates of a psychiatry CE track.^18^ However, survey responders also remarked on several less intuitive benefits, such as increasing resident satisfaction with the residency program. The underlying perception that the program supports residents' long-term career goals may affect its reputation among its residents. CE tracks can also strengthen resident recruitment efforts. This benefit was previously suggested when a family medicine program noted a greater volume of applicants and higher measures of applicant quality after introducing a CE track.^6^ Survey responders also report that a CE track can improve the quality of neurology education in the larger environment of the department, for example, concretely in educational sessions targeting an audience beyond their peers or more abstractly promoting an environment of continued learning. The results of our study suggest that the benefits of a CE track extend beyond neurology residents themselves with downstream beneficial effects on the residency program and the department. We feel the downsides to implementing a track are few. However, 1 concern is that residents who do not participate in a CE track may not have the same opportunities to engage in education training as residents who are enrolled in the track. This may be especially relevant for residents who develop an interest in becoming CEs late in residency. While the authors are not aware of studies that have assessed this risk, programs should be cognizant of providing medical education opportunities to all residents who are interested, regardless of whether they matriculate to a CE track.

Support for developing or maintaining a CE track may also be available outside the institution. Our study suggests that one impactful intervention would be the development of live or recorded lectures for residents on medical education topics, reducing the need for individual faculty time. An example is the resident as educator training program, run through the American Academy of Neurology (AAN), a longitudinal 1-year program focusing on core educational concepts.^19^

Requirements for track participants were similar across programs, although there was great variability in the resources available to participating residents. This finding could be related to the different levels of resources available to programs and institutions but could also relate to a lack of national guidelines around CE tracks. Prior research has recommended certain CE track programmatic attributes, including protected time and funding for participants, availability of a network of mentors, incorporation of multiple instructional methods focusing on experiential learning, and clear and defined goals and objectives.^10^ While 100% of survey respondents with CE tracks reported providing mentorship, only about half provide funding for a scholarly project and less than half provide protected time or funding for conference attendance, indicating that there are actionable areas where programs can bolster their CE track. Although this study did not ask about specific topics taught in tracks, prior scoping reviews of CE tracks in GME found that teaching skills and medical education scholarship were the most common topics taught while leadership, administration skills, and career development were less common but still included in greater than 50% of CE track curricula.^9,10^ For programs looking to either implement or enhance a CE track, the publication of the CE milestones is a helpful guide.^3^

Our study had several limitations. Our response rate of approximately one-third of US adult neurology residency PDs could limit generalizability, with the survey selecting respondents who already have CE tracks in their programs. Similarly, PDs without CE tracks who responded may be more likely to report considering developing a track than those who did not respond. The geographic distribution of our survey results was largely similar to the distribution of neurology residency programs. However, there was an over-representation of respondents in the Middle Atlantic geographic region (30% vs 19.5%) and an under-representation of respondents from the Mountain West (1.8% vs 5.6%).^16^ We also did not include child neurology residency programs. In addition, 1 PD emailed the authors indicating that they specifically recruit applicants interested in medical education, and because they provide medical education didactics all year for all their residents and their resident class is small, the survey did not feel relevant to their program. There may have been other PDs who did not respond to the study because of a similar concern. The email highlights that a CE track may not be necessary for all programs, especially if they have a robust resident-as-teacher curriculum targeted to all residents. However, the numerous benefits identified in our study suggest that for most programs, a track would benefit interested residents and the program.

Our study suggests that adult neurology PDs and other education leaders endeavoring to develop or enhance a CE track have ample justification. Most of the PDs agree that CE tracks are invaluable in helping neurology residents develop as teachers and future education leaders. The majority also agree that the benefits of CE tracks extend beyond the participants, contributing to the health and reputation of the residency program and the neurology department. The heterogeneity among CE tracks suggests that less robust programs could introduce more elements to bolster their effectiveness and become more competitive with peer programs. Cross-institutional resources, such as those championed by the AAN, could also relieve the resource burden on individual programs. For programs that decide a CE track is not necessary, they may still benefit from incorporating centralized CE resident resources into their didactic curriculum. As the concept of the CE role evolves, it will be important that efforts to enhance CEs' professional development are appropriately funded and resourced because these investments may have far-reaching benefits.

## Appendix Authors

**Table:**

Name	Location	Contribution
Nuri Jacoby, MD	Department of Neurology, SUNY Downstate Health Sciences University; Department of Neurology, Maimonides Medical Center, Brooklyn, NY	Drafting/revision of the manuscript for content, including medical writing for content; major role in the acquisition of data; study concept or design; analysis or interpretation of data
K.H. Vincent Lau, MD	Department of Neurology, Boston University School of Medicine, MA	Drafting/revision of the manuscript for content, including medical writing for content; major role in the acquisition of data; study concept or design
Maureen I. Ekwebelem, BS	Division of Geriatrics and Palliative Medicine, Weill Cornell Medicine, New York, NY	Major role in the acquisition of data
Jeremy J. Moeller, MD, MSc, FRCPC	Department of Neurology, Yale School of Medicine, New Haven, CT	Drafting/revision of the manuscript for content, including medical writing for content; study concept or design
Daniel Shalev, MD	Division of Geriatrics and Palliative Medicine, Weill Cornell Medicine, New York, NY	Drafting/revision of the manuscript for content, including medical writing for content; study concept or design; analysis or interpretation of data

## Glossary

AAN: American Academy of Neurology
ACGME: Accreditation Council for Graduate Medical Education
CE: clinician-educator
GME: graduate medical education
PD: program director
